# Explicit motives and personality characteristics in first year medical students: a multicentre quantitative study using McClellands motive disposition theory

**DOI:** 10.1186/s12909-024-05717-3

**Published:** 2024-07-12

**Authors:** Johanna Flora Rother, Michelle Seer, Stephan Stegt, Tobias Raupach

**Affiliations:** 1 Institute of Medical Education, University Hospital Bonn, Venusberg- Campus 1, 53127 Bonn, Germany; 2 Institute for Test Development and Talent Research, ITB Consulting GmbH, Bonn, Germany

**Keywords:** Motivation, Explicit motives, Emotional intelligence, Empathy, Personality, Gender differences, Medical students, Medical education

## Abstract

**Background:**

Despite the challenging curriculum, medicine is a popular study program. We propose McClelland’s Motive Disposition Theory (MDT) as a possible theory for explaining medical students motivation. The theory describes how individuals differ in their behaviour due to their varying manifestations of certain motives. The three motives can thus influence the students behaviour and academic success. Using these motives, complimented with an altruism- and a freedom motive, this study was aimed at investigating young adults’ explicit motives to study medicine. In addition, we also wanted to find out whether there are gender differences in motives and other variables such as empathy, emotional intelligence and academic self-concept.

**Methods:**

Over 20 universities across Germany were contacted and asked to share the online study with their first semester medical students in the winter term 2022/23, which resulted in a final *N* = 535. We used validated and reliable measurements, including a self-created and piloted questionnaire covering medicine-specific explicit motives.

**Results:**

Comparing the mean scores between motives, we found that the altruism motive was the strongest motive (*M* = 5.19), followed by freedom (*M* = 4.88), affiliation (*M* = 4.72) and achievement (*M* = 4.59). The power motive achieved the lowest score (*M* = 3.92). Male students scored significantly higher for power (*M* = 4.24) than females did (*M* = 3.80, *p* < .001), while female students found affiliation more important (*M* = 4.81) than male students did (*M* = 4.59, *p* = .016). Female participants scored significantly higher for emotional intelligence (*p* = .010) and several personality aspects, including empathy (*p* < .001), but showed a significantly lower academic self-concept (*p* = .033), compared to their male colleagues. Nonetheless, the effect sizes were mostly small to medium.

**Conclusions:**

Our findings suggest that first-year medical students are primarily motivated by humanitarian factors to study medicine, compared to motives related to money or power. This is mostly in line with earlier studies using qualitative approaches, showing that MDT can be applied to explain explicit motives in medical students.

**Trial registration:**

The longitudinal project, which this study was part of, was registered via OSF (https://archive.org/details/osf-registrations-mfhek-v1) on the 28th of September 2022 under the title “Transformation of emotion and motivation factors in medical students during the study progress: A multicenter longitudinal study”.

## Background

Medicine is one of the most popular study programs worldwide, despite the challenging curriculum. While other study programs often struggle with high dropout rates up to over 40% [1], dropout rates for medicine are relatively low [2]. One possible reason for the low dropout rate in medical students, in addition to the good career prospects, is the high intrinsic motivation in medical students [2]. Intrinsic motivation describes a person’s inner values or interests that drive them to engage in certain behaviours. This differs from extrinsic motivation, where the behaviour is rewarded or punished by external factors such as money or evaluation through others [3]. Higher intrinsic motivation in students does not only lead to lower dropout rates, but is also associated with several positive outcomes such as improved well-being and academic success [4]. Therefore, understanding medical students’ motivation and facilitating intrinsic motivation seems essential to ensure consequences such as successful graduations and careers, especially in a demanding specialty such as medicine. When examining motivation in medical students, it seems essential to assess the motivation of the students in the context of which stage of their education they are currently in. In a literature review, Kusurkar and colleagues [5] summarize that most medical students start their education and chose their profession based on intrinsic goals and autonomous motivation. Since then, several recent studies have discovered higher levels of intrinsic motivation in earlier stages of their medical education, compared to students who were further in their education [6, 7]. The authors hypothesized that, along with other factors, the strict and discipline based curriculum might contribute to some of these motivational shifts [7]. Other studies found that motivation in medical students also differed depending on school form and gender [8]. Therefore, motivation seems to not only be an independent variable in medical education due to the influence on several important learning outcomes, but also a dependent variable, which can be influenced itself [5].

For medical students, several studies [5, 9] use the *Self Determination Theory* (SDT) [10] to explain motivation. This seems like a valid approach, since the subdimensions of motivation based on the SDT seem to change throughout the educational process [7]. This theory postulates that fulfilment of the three needs competence, autonomy and relatedness enhances intrinsic motivation. Another theory to explain motivation is the *Motive Disposition Theory* (MDT) [11]. This theory includes the three motives affiliation, power and achievement and states that individuals with different learning experiences differ in their needs, depending on how strong each motive manifests [3, 12]. Therefore, different individuals experience and evaluate the same situation differently, depending on how well the situation fits their preferred motive. This allows between-subject differences in variables such as motivation, since each person has an individual predisposition for the motives, which differs from the SDT. Consequently, we chose the MDT as a theoretical framework, since our aim was to examine differences between individuals in different study environments in the context of a longitudinal story, which will be explained later on. Similar to motivation, motives have also been specified and separated into the concept of implicit and explicit motives [13]. Explicit motives were chosen for this study since they predict an individual’s chosen behaviour in a more structured situation where a person is aware of their motives and goals [3, 13] such as studying and they can be assessed more easily trough questionnaires.

Most of the existing studies that examine motives in medical students used qualitative measurements and did not systematically assign the answers to motive categories [14, 15] or measured their general amount of motivation [16]. In a German study, medical students in different stages of their study found job related factors to be most important, such as the spectrum of specialties within medicine. The other important aspects were mostly humanitarian or scientific interest [14]. Interestingly, the less advanced students were more interested in altruistic factors and a good balance between their career and their personal life than more advanced students [14]. A systematic review by Goel and colleagues [17] found that students from higher income countries, such as Germany, selected scientific and humanitarian factors as their main motivators, while students from lower to middle income countries mostly reported societal factors, such as social status or job security. The authors refer to Maslow’s need hierarchy [18] and argue that while participants from lower income countries need to satisfy their basic needs of safety and security, participants from higher income countries have the privilege of focussing on more complex needs, such as scientific interest or self-actualization. Self actualization sits on top of the needs hierarchy and, according to Maslow, describes a state when an individual reaches its full potential, making it the ultimate goal [17]. Studies have also shown differences between men and women in their preferred motives. While men showed higher scores for societal factors such as a good reputation and good career opportunities, women preferred humanitarian and altruistic motives such as responsibility or helping patients [14].

To further examine the cohort of medical students and find out whether male and female students differ in other aspects, we also wanted to assess career-related personality characteristics. Using personality assessments as an additional variable in the admission process in medical schools has been discussed previously [19], making them a relevant variable. To include these in our examination of first year medical students, we included personality characteristics such as emotional intelligence and empathy, which are important for many medical professions, to see whether there were any significant gender differences.

Our last variable of interest was the academic self-concept. Academic self-concept refers to the perception of individuals about their abilities and their own academic achievements [20]. It has not received as much attention in medical education as other constructs, such as academic self-efficacy [21]. Studies have found that it not only affects several academic behaviours, such as engagement or motivation, but also that a higher academic self-concept in medical students was related to academic success in the preclinical and clinical phase [21]. A cross-sectional study with two cohorts of medical students in the USA has shown gender differences, with men showing higher academic self-concept than women [21]. This possibly relates to findings that men are more confident in their abilities than women are and have a stronger sense for competition [22], which might put male students at an advantage in testing and exam situations [23]. Therefore, we wanted to examine whether this phenomenon of higher confidence was also the case for German first year medical students and hypothesized that, based on previous research, male students have a stronger academic self-concept than female students do. Since earlier studies showed a clear picture of men having a higher academic self-concept, we chose a directional hypothesis to examine this variable due to the increased statistical power.

In summary, we wanted to investigate which motives drive medical students to study medicine and how their personality, emotional intelligence and academic self-concept manifests at the beginning of their education. In addition, we were interested in possible gender differences. Therefore, our research questions and hypothesis are as follows:

### RQ1

What drives first-year students to study medicine?

### RQ2

Do different genders have different motives to study medicine?

### RQ3

Do different genders of first-year medical students show differences in their (career-related) personality traits?

### H3.1

Male medical students have a significantly higher academic self-concept than female medical students.

## Methods

This study was part of a longitudinal research project called ‘**T**ransformation of **E**motional and **M**otivational factors in **M**edical **S**tudents (TEMMS)’ which aims at exploring possible changes in students throughout undergraduate medical education and how the different structures of study programs in Germany influence this transformation. The study involves annual data collections from multiple sites within Germany. The data presented here were obtained in the first wave of the survey. As a consequence, these data do not facilitate any inferences on the transformation of medical students over time. The TEMMS study was approved by the local Ethics Committee (application number 2022-342-BO).

### Participants

In the summer of 2022, we contacted every german public university with a medical study track and asked them to share information about the study in their first-year medical courses in winter term 2022/23. In order to investigate the structure of the study programms for the TEMMS project, we focussed on public universities and did not contact private universities. Out of the 38 contacted universities, 25 agreed to share the materials with their students via mail servers, notification on the study platform or via members of the student council. Participants received links and QR-codes to access the online survey, which was conducted via Limesurvey. After giving their informed consent, they were asked to complete the questionnaire on their personal device and on their own time, which took about 40 min. 1199 students students started the questionnaire, of which we excluded 664 since they either did not finish the questionnaire, were enrolled in a later semester than the first or indicated that they studied a track other than human medicine, such as dental medicine. The final sample therefore consisted of 535 first-year medical students from 22 different universities across Germany with complete answer sets. The mean age was 20.6 years (*SD* = 2.9), ranging from 16 to 34. Of all participants, 374 participants were female, 151 were male, one diverse and nine did not indicate their gender. The gender ratio in our sample aligns with the gender ratio in German medical students, since almost two thirds of this population were female in the year 2022 [24]. The sample size at individual universities ranged from four to 80 (*M* = 24.3). There were no rewards (such as class credits) given to the students, but they were offered to receive a breakdown of their results in the personality questionnaire.

### Variables and instruments

We used various questionnaires in the TEMMS study, but since not all of these instruments are relevant for this first assessment, only some questionnaires will be described in detail. Firstly, demographics such as gender, university, course program, year of study and previous experience in the medical field, such as internships and work experience were assessed. Upon completion of the questionnaire, participants were asked whether they consented to provide their email address to be contacted for the next surveys in the longitudinal project. To ensure anonymity, they were redirected to a different site to enter their email address.

### Motives

Since there was, to our knowledge, no validated questionnaire to measure the motives in medical students based on the MDT, a questionnaire was created and piloted. In addition to the three motives achievement, affiliation and power stemming from the MDT, to which Kuhl [25] has added the motive of freedom in his PSI-Theory [26], we wanted to include a motive which relates to the humanitarian factor of a medical profession and captures the social aspect of the patient contact. Therefore, in cooperation with three external psychologists with experience in the motivational field, one physician with experience in the assessment of motives in medical students and three other psychologists from our institute, we added an altruism motive. Compared to the affiliation motive, altruism does not rely on a connection with the other person to explain the need to help and care for someone. Therefore, the affiliation motive describes the connection to colleagues, while altruism describes the pure need to help others. The items for this instrument were identified and summarized from multiple studies over the last 20 years and each assigned to one of the five motives by a group of experts. In the pilot study, using a sample of 190 medical students which were enrolled at the University of Bonn, the instrument was then compared to the factorial structure of validated questionnaires (IMPART, GOALS-questionnaire). The goal was to determine whether an explorative factoral analysis would detect a similar five factoral structure in our questionnaire, which would align with the validated questionnaires. We found a good fit for the five factorial structure, with TLI = 0.913, CFI = 0.934 and RMSEA = 0.052. Internal consistency mostly ranged from 0.758 to 0.827, with the exception of freedom. Despite this lower reliability, the authors decided to integrate this factor since development of the model content takes precedence over the statistical confirmation of the model. We identified the items with the highest factoral lodings and only included items over 0.3. A maximum likelihood with varimax-rotation afterwards revealed an explained variance of 49.7%, which came close to the 51.7% which were found for the compared questionnaires surrounding the general motives. The final questionnaire consisted of 24 medicine-specific items, with five items for the subscales achievement, power, freedom and altruism and four items for the affiliation subscale. Participants rated the items on a scale from one (“Not at all important to me”) to six (“Very important to me”), resulting in a mean score from one to six for each motive, with a higher score indicating a higher importance assigned to the motive.

### Emotional intelligence

To measure emotional intelligence, we used the non-linguistic *Face-Based Emotion Matching Test* (FEMT) [27]. Participants were presented 18 image pairs of different faces. While looking at the pictures, they had to decide whether the faces showed the same, rather same, rather different, different expressions or whether they did not know. Participants only received a point when they identified the same or different emotion correctly and did not receive points for “rather the same“/“rather different“, or the “I don’t know“ option. Therefore, participants could achieve a score from zero to 18, with higher scores indicating higher emotional intelligence. It was chosen due to the objectiveness of the instrument, compared to self-reports of emotional intelligence. A multistudy report, which examined the psychometric properties of this instrument using several samples between 182 and 383 participants, demonstrated an acceptable goodness of fit, normal distribution of scores and acceptable Cronbachs αs. The items have also been shown to be sample- and gender-invariant [27].

### Personality

Personality characteristics were assessed using the German *ITB-PESA Personality Structure Assessment* [28], which is used to measure job-relevant characteristics and is based on several psychological constructs, including the *HEXACO*-Personality model [29]. The instrument avoids classifications into different types or categories and provides a modular-scale system with 23 scales total to provide seperate assessment of different personality aspects [28]. The instrument has been validated first using a sample of 398 students, later using a sample of 405 students. Internal consistency was above 0.70 for 20 of the 23 scales, with three ranging between 0.60 and 0.70 [28]. Using the HEXACO-PI-R showed high correlations between similar PESA and HEXACO scales and low correlations with different scales. For the specific scales we employed retest reliability ranged between 0.61 and 0.91 [28]. It was chosen mainly due to the fact that the items specifically refer to career-related situations, whereas other personality tests usually refer to every-day situations. We chose seven out of the 23 scales, based on relevance for our medicine-specific context. The seven scales were *Outgoingness, Open-mindedness and curiosity, Consensus orientation, Empathy, Perseverance and the ability to cope with pressure, Rule-consciousness* and *Honesty* with 66 items in total. Every scale had between eight and ten items and each item was rated on a six-point Likert-scale from one (“Do not agree at all”) to six (“Completely agree”). A sum score was calculated for each scale, with different ranges according to the different item counts for each scale. A higher score indicates a higher manifestation of the personality trait.

### Academic self-concept

To assess the academic self-concept and the perception of the students of their own abilities and intelligence, one of the *Academic Self-Concept Scales* [30] was administered. The participants had to rate each of the five items on a scale from one (“low”) to seven (“high”), from which a mean score was derived, with higher scores indicating a higher academic self-concept. The specific scale we used measures academic self-concept without referring to an explicitly given norm [30] The instrument has university-student specific items, and has been validated using three samples of german students, showing high validity and good internal consistency [30].

### Data analysis

IBM SPSS Statistics (Version 27) was used to analyse the data. Firstly, the data set was prepared and mean and sum scores for the variables were calculated. To compare the motives, we compared the mean value for the motives. To examine possible gender differences in the motives, the emotional intelligence, the personality characteristics and the academic self-concept, unpaired t-tests were conducted.

## Results

**Fig. 1:**
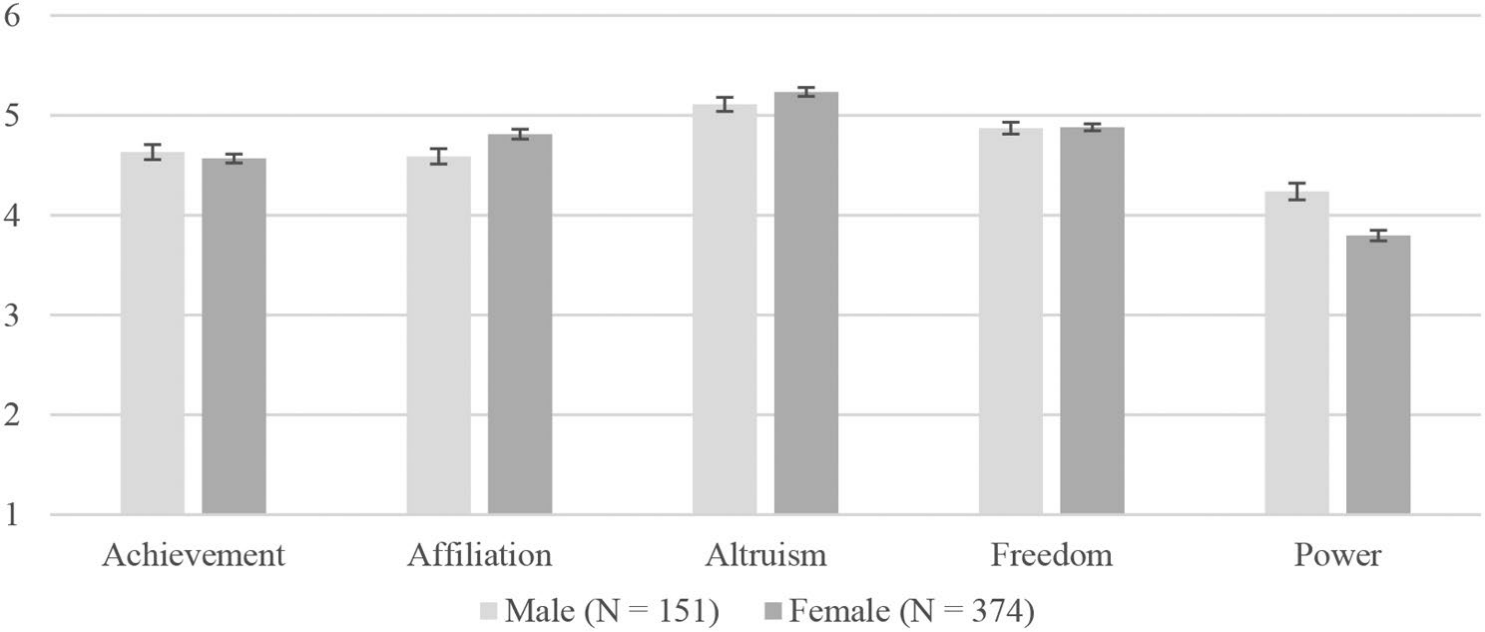
Explicit motives in female and male medical students. *Notes*. Means and standard errors

In the overall sample, the motive of altruism was ranked the highest (*M* = 5.19). Freedom (*M* = 4.88) was in second place, while affiliation (*M* = 4.73) followed. Achievement (*M* = 4.59) and power (*M* = 3.92) were ranked least important. T-tests for independent samples revealed some significant differences between male and female medical students. While male students rated power as more important (*M* = 4.24) than the female students (*M* = 3.80, *t*(523) = 4.47, *p* < .001), women had higher rankings for affiliation (*M* = 4.81) than male participants (*M* = 4.59, *t*(523) = -2.42, *p* = .016). An overview of the means and standard errors for the motives can be found in Fig. 1.

With regard to emotional intelligence, students in the full sample obtained a mean score of 11.2 (*SD* = 2.9), translating to a mean percentage of 62.2% (*SD* = 16.1%) of pairs identified correctly. Women (*M* = 63.4%) scored significantly higher than men did (*M* = 59.4%, *t*(523) = -2.59, *p* = .010), even though Cohen’s *d* only revealed a small to medium effect size of − 0.25.

There were several differences between male and female participants in the seven personality scales of the PESA-questionnaire. Women scored significantly higher on empathy (*t*(523) = -7.64, *p* < .001), consensus orientation (*t*(523) = -4.04, *p* < .001), honesty (*t*(523) = -3.95, *p* < .001) and rule-consciousness (*t*(523) = -3.43, *p* = .001). An overview of the mean scores can be found in Table 1. Regarding the gender differences, it has to be noted that the sample sizes for men and women were fairly unequal, which can be found in Table 1. Nonetheless, the variables where the differences reach significance are marked.

**Table 1:** Means and standard deviations of the relevant variables

Variable	Male (*N* = 151)		Female (*N* = 374)		Complete Sample (*N* = 535)	
	M	SD	M	SD	M	SD
Achievement	4.63	0.92	4.57	0.86	4.59	0.88
Affiliation*	4.59	0.95	4.81	0.96	4.72	0.97
Altruism	5.11	0.86	5.23	0.85	5.19	0.87
Freedom	4.87	0.72	4.88	0.67	4.88	0.68
Power*	4.24	1.03	3.80	1.02	3.92	1.04
EI (in %)*	59.42	15.53	63.41	16.17	62.15	16.07
Empathy*	21.15	6.84	25.85	6.20	24.45	6.74
Consensus orientation*	29.66	7.28	32.29	6.54	31.59	6.83
Honesty*	30.34	8.50	33.32	7.50	32.40	7.92
Open-mindedness	33.17	4.98	33.80	5.29	33.61	5.18
Outgoingness	24.62	8.50	25.90	8.47	25.56	8.42
Perserverance	34.30	7.91	34.20	7.59	34.21	7.62
Rule-consciousness*	27.25	6.78	29.45	6.61	28.76	6.69
ASC*	5.22	0.77	5.07	0.86	5.11	0.84

EI = Emotional Intelligence, ASC = Academic Self-concept, * = *p* < .005

The mean score for the academic self-concept in the sample was 5.11 (*SD* = 0.84). As expected, men scored higher than women (*t*(518) = 1.84, *p* = .033), even though Cohen`s *d* only revealed a small effect of − 0.18.

## Discussion

We shared an online survey with over 20 medical schools across Germany and examined explicit motives, personality characteristics and emotional intelligence in 535 first-year medical students, using validated questionnaires. After comparing the ratings for the motives, which were adapted from the motive disposition theory [11] and complimented with two other motives, we found that both men and women reported altruistic motives, such as helping people and doing good, as their primary reason to study medicine. Men ranked power as more important than women, while women had higher ratings for the affiliation motive. Female participants had significantly higher scores for emotional intelligence and several personality characteristics, such as empathy, while male participants showed a higher academic self-concept than females.

Since this -to our knowledge- is the first study that systematically studies motives in this generation of medical students using the MDT by McClelland [11], we can only relate these findings to studies using different data collection tools. These studies examined specific reasons to study medicine not with the use of a motivational theoretical background, but rather by sorting them into categories, such as humanitarian or scientific [17] or intrinsic and extrinsic [31]. Looking at our results regarding the importance of the altruism motive for first-year medical students, our results are in line with similar findings where prospective Generation Z medicine students (born 1995 to 2010) reported helping people as a core motivation [9] and findings that especially first-year students rated helping people as more important than more advanced students did [14]. It also fits with earlier mentioned findings where students in earlier stages of their education were more motivated by intrinsic factors and less motivated by external factors, which seems to align with our findings regarding the external motivation through power. Therefore, this sparks the question whether this will also change over time.

Interestingly enough, our finding that freedom was the second most important motive also compares to data reported by Becker et al. [14], where less advanced students also rated good compatibility of family and work as more relevant than more mature students, which raises the question whether perceived relevance of this aspect declines over time.

Since power was the least important motive for men and women, this strengthens Goel and colleagues’ [17] assumption that young adults from higher income countries have the luxury to focus on self-actualization related goals, referring to Maslow’s need hierarchy. Our findings are in line with this hypothesis. In a lower-income country, this study might have produced different results regarding the power motive, since students there have to focus on fulfilling their primary needs and providing for their family [17]. Since the power motive includes societal status and in this context, the desire for a higher salary, the need to provide could possibly manifest through a higher power motive.

As mentioned earlier, Becker et al. [14] found that female students rated the caring aspects of the medical profession as more important, while male students put more emphasis on career-related aspects. This mostly aligns with the gender differences we found in our study. Since a recent survey revealed that many young men in Germany still have a traditional image of role distribution in relationships and see themselves as the main earner of the family [32], this could explain the significant difference in the need for power. Nonetheless, it is interesting to note that while men had higher rankings for the power motive, the predicted differences for the caring and social aspects did not manifest themselves in the altruism motive, but rather in the affiliation motive. Consequently, women seem to value working in a team and building good relations more than men do, which manifests in the higher affiliation motive, while the altruistic aspects of the medical study track seem to be equally important for both genders. This differs compared to previous studies.

The strong altruism motive also seems medicine-specific. To name one example, Jacobsen and Diseth [33] found that psychology students primarily chose their course out of interest in the subject and to increase their understanding of themselves and others, which suggests that medicine might still be more popular when it comes to choosing a study track in order to help people further on.

Women scored higher in several personality aspects, such as empathy, which is in line with earlier findings regarding empathy in medical students [34, 35] and emotional intelligence [36], even though the effects were only small. Since previous research has shown significant changes in empathy and emotional intelligence throughout undergraduate medical education [37], it will be interesting to see whether the present longitudinal study finds similar transformations.

The predicted gender difference between female and male students in academic self-concept, which was found in earlier studies [21], only showed a small effect size. It will also be interesting to see whether the longitudinal study shows possible changes in this variable and in the gender difference. The difference also hints at a need for improvement of academic self-concept in female students as well as male students with particularly low values, since the lack of confidence could have negative consequences on their academic achievements [21].

### Limitations

There are some limitation to this study. The most important limitation is the lack of a comparison group of the results regarding the motives. Since we did not examine other study courses, we cannot draw any conclusions about whether these motives are different in other study tracks and can only vaguely compare our findings to previous studies. Therefore, this study does not give insight about whether medical students have a particularly strong altruism motive, but it shows that medical students see altruism and freedom as more important in their future career than, for example, achievement, despite some gender differences. The same applies for other variables such as emotional intelligence or academic self-concept, since we cannot compare our results with data obtained from students of other programs. Consequently, additional data is needed in order to put these results into a broader perspective. Since this study was only the first assessment of a longitudinal study, further results will give more insights about possible transformations in these variables.

Additionally, we only examined explicit motives. Since these were assessed through self-report, the social desirability bias might affect the results. Even though this should not be an issue due to the anonymity of the study, we cannot rule it out as a possible explanation. Especially when it comes to altruism, students might have rated it higher to maintain a positive image and to give the impression of a good person. In future studies, this should be addressed and avoided, for example with the use of control items. In the same vein, research has shown that while caring and communatal traits are typically expected of women, motives such as power and ambition are stereotypically expected in men [38]. Therefore, this could influence the results since explicit motives are controlled voluntarily and could be adapted based on gender based expectations. Regarding the gender differences, it is important to mention that similar to the gender variance in medical students, almost 70% of our sample was female. Therefore, this might have affected the statistical difference and hinders the generalization of the results.

In addition to the sample size, one limitation regarding the missing gender difference in altruism, which has been shown in earlier studies [14] might be a possible ceiling effect. Since both genders’ mean values were above 5.1 [1:6], possible differences might be harder to detect. In summary, it would be interesting to examine implicit motives to see whether these might differ from the results on explicit motives and whether these limitation could be avoided.

## Conclusion

In summary, our findings regarding the importance of motives are in line with earlier studies for comparable cohorts in similar cultural contexts and show that the motive disposition theory can also be applied to medical students in order to examine their motives to study medicine. Even though female and male students differed in some aspects, which is in line with earlier findings regarding gender stereotypes and personality differences, altruistic motives seem to be most important for first-year medical students. Circling back to the intrinsic motivation in medical students, the theory states that intrinsic motivation is reached when a person’s individual basic need is satisfied. After identifying altruism as a core motivation in students, the students should be even more motivated when they are able to actually get to the practical part of their education, for example through residency and internships. Therefore, it will be interesting to see whether the altruistic motive holds its relevance or whether confrontation with real patients changes the students’ attitude. Applying these results in medical education might also be helpful in order to ensure that the students motivation does not shift negatively as it has been seen before [7]. Since first year students show higher altruistic motives and are less motivated by external factors, it seems beneficial to consider the factors which lead to such motivational change. Since altruisms seems like a valuable quality to have in medical professionals, further studies should investigate how interventions could promote altruism in further stages of the study program. Due to other authors proposals that the strict and traditional curriculum might play a role in this change [7], it could be beneficial to consider these results when talking about curriculum changes.

Since we assessed the motives after a few weeks and months into the undergraduate curriculum, this mostly describes what drives medical students to choose their study course. The longitudinal project will be able to detect whether important aspects of the curriculum, such as the first exams or the first contact with patients will impact these motives. Nonetheless, we successfully applied a motivational framework to assess motivation in medical students. Future studies could compare these results to other study tracks, such as psychology or even other forms of healthcare studies, such as dentistry.

## Data Availability

The datasets used and/or analysed during the current study are available from the corresponding author on reasonable request.
